# Establishment of immortalized primary cell from the critically endangered Bonin flying fox (*Pteropus pselaphon*)

**DOI:** 10.1371/journal.pone.0221364

**Published:** 2019-08-26

**Authors:** Tetsuya Tani, Takahiro Eitsuka, Masafumi Katayama, Takashi Nagamine, Yumiko Nakaya, Hajime Suzuki, Tohru Kiyono, Kiyotaka Nakagawa, Miho Inoue-Murayama, Manabu Onuma, Tomokazu Fukuda

**Affiliations:** 1 Laboratory of Animal Reproduction, Department of Agriculture, Kindai University, Nara, Japan; 2 Graduate School of Agricultural Science, Tohoku University, Sendai, Japan; 3 Wild life Genome Collaborative Research Group, National Institute for Environmental Studies, Tsukuba, Ibaraki, Japan; 4 Ecological Risk Assessment and Control Section, Center for Environmental Biology and Ecosystem, National Institute for Environmental Studies, Tsukuba, Japan; 5 Conservation & Animal Welfare Trust, Maehara, Uruma, Okinawa, Japan; 6 Institute of Boninology, Chichijima, Ogasawara, Tokyo, Japan; 7 Division of Carcinogenesis and Cancer Prevention and Department of Cell Culture Technology, National Cancer, Center Research Institute, Tokyo, Japan; 8 Wildlife Research Center, Kyoto University, Kyoto, Japan; 9 Graduate School of Science and Engineering, Iwate University, Morioka, Iwate; 10 Soft-Path Engineering Research Center (SPERC), Iwate University, Morioka, Iwate, Japan; Kansas State University, UNITED STATES

## Abstract

The Bonin flying fox (*Pteropus pselaphon*) is one of the most critically endangered species of animals. The number of this species is estimated to be around 150; being classified at the top rank in the list by International Union of Animal Conservation. Our group previously showed that expression of CDK4, CYCLIN D1, and telomerase reverse transcriptase (TERT) efficiently induce immortalization of human, bovine, swine, monkey, and buffalo-derived cells. In this manuscript, we successfully established the primary cells from Bonin flying fox. We introduced CDK4, CYCLIN D1, and TERT into the primary cells. The established cells showed efficient expression of introduced genes at the protein level. Furthermore, the established cells were free from senescence, indicating it reached to immortalization. Moreover, we showed that interspecies somatic cell nuclear transfer of Bonin flying fox derived cell into bovine embryo allowed the development of the embryo to 8 cell stages. Our established cell has the potential to contribute to species conservation.

## Introduction

The Bonin flying fox (*Pteropus pselaphon*) is an original species in North and South Iwo Jima, the regions of Bonin Islands of Japan. During World War II, Bonin flying fox was hunted and exported as military food to Guam Island. Due to the hunting practices for food purpose, and forest destruction by human activity, the number of Bonin flying fox species significant decreased around the early 1970s. Bonin flying fox was believed to have reached extinction, since there was no report on the evidence of its survival. However, in 1986, a colony of Bonin flying fox was found in a cave of Chichijima, one of the Bonin Islands. Consequently, with adequate protection and conservation activity, Bonin flying fox was nominated as the “Natural monument” of Japan in 1969, and classified as an original endangered species in 2009. Furthermore, the International Union for Conservation of Nature (IUCN) ranked as the most critically endangered animal, with the highest risk to extinction. Presently, the estimated number of Bonin flying foxes is around 130–150.

In our previous studies, we showed that the expressions of mutant cyclin dependent kinase (CDK4) and CYCLIN D1, and telomerase reverse transcriptase (TERT) induce the immortalization in multiple species [1–7]. Since the amino acid sequence of CDK4 and CYCLIN D1 is conserved in the process of evolution from yeast to humans, this immortalization method might apply to a wide variety of species. Furthermore, the function of p53, one of the critical gate-keeping genes needed to maintain the intact genome, is still integral in these cell lines. We reported that cells immortalized with this method retain the original chromosomal pattern as well as the nature of primary cells [6,8,9]. Due to the intact chromosomal condition, we speculate that the re-generation of Bonin flying fox might be possible if we apply the somatic cloning technique to immortalized cells. As the representative cellular immortalization methods, expression of SV40T [10] or TERT [11], or a combination of knockdown of p16 and c-MYC have been reported [12]. We need to select the best method for the immortalization, which depends on the cell and species specificity.

As the bat derived cells, the establishment of primary cells and cellular immortalization with TERT or SV40 large T expression was previously reported [13,14]. The establishment of the megabat derived cells were worth for the investigation since megabats mediate various types of infectious diseases, such as Nipah and Hendra viruses. The establishment of Bonin flying fox derived cells allows us the detailed genome analysis, which would help to understand how megabats had made diversity on the animal evolution.

It is noteworthy that primary cells reach cellular senescence after a certain number of cell division cycles, resulting in the halt of cell proliferation. In the process of the cellular senescence, p16 protein is known to accumulate in the cells. This protein binds to the specific site of CDK4 and down-regulates the enzymatic complex of CDK4-CYCLIN D1. Loss of CDK4-CYCLIN D1 activity results in hypo-phosphorylation of Retinoblastoma protein (RB), which results in the halt of the cell cycle. However, we previously showed that the expression of R24C mutant CDK4 and its counterpart protein, CYCLIN D1, TERT efficiently accelerate the cell growth speed. The point mutation of 24^th^ amino acid of CDK4 causes the structural change of the binding site of p16 and resulting in a constitutive active status of CDK4 kinase, which is free from negative regulation of p16 [9]. There are several ways to bypass the negative effect of p16 protein on cell growth, such as knockdown of p16, or expression of R24C mutant CDK4. Bypass of senescence protein p16 is quite critical to overcome the limitation of cell growth.

These situations indicate that the primary cells are a limited resource. These situations guided us to build the hypothesis that the expressions of mutant CDK4, CYCLIN D1, and TERT would allow us to establish the immortalized cells from Bonin flying fox. The establishment of Bonin flying fox cell lines will allow us to extract high molecular and quality genomic DNA infinitely. We can preserve the cultured cells, which are crucial to obtain the sequencing information of the genome and or total RNA. 　The establishment of Bonin flying fox-derived immortalized cells would contribute to understanding the diversity and branching out of the Bonin flying fox species from other is megabats during evolution.

## Materials and methods

### Primary cells of Bonin flying fox

We described the detailed information and history how the tissues of Boninflying fox we obtained in the section of Ethics Statement. We collected the tissues of 5^th^ finger of the right-wing and skin and transferred into the National Institute of Environmental Studies in Tsukuba. We maintained the primary cells in the DMEM/F12 medium (Wako Chemical, Osaka, Japan) containing 10% fetal bovine serum (FBS) and 1 X antibiotics mixture (Nakarai Tesque, Kyoto, Japan). We preserved the expanded cells in liquid nitrogen tank with 10% dimethyl sulfoxide (DMSO) medium until use. We carried out the surgical procedure and handling based on the Act on Welfare and Management of Animals (Act No. 105 of October 1, 1973).

### Preparation of recombinant viruses for cellular immortalization

For the preparation of lentivirus, we obtained all related plasmids were from Dr. Hiroyuki Miyoshi (RIKEN, BioResource Center, Tsukuba, Japan). We introduced the packaging plasmids, pCMV-VSV-G-RSV-Rev, pCAG-HIVgp, CSII-CMV-EGFP (total 6μg) into 293T for the transient packaging with lipofection. The virus supernatant was recovered and filtered with 0.45mm disk filter (code 17598, Sartorius AG, Göttingen, Germany), and we carried out the concentration of recombinant viruses with centrifuge and precipitation under the existence of PEG6000. The concentrated virus pellets from 30ml of supernatants were resuspended with 1.5ml of culture medium, and the viruses were used for the infection to the target cell with the existence of 4μg/ml of polybrene for 48 hours. We previously described more details about the protocol to obtain the high titer virus [15].

For the preparation of Moloney Murine Leukemia Virus (MMLV) retrovirus, or we used QCXIN-CDK4R24C-P2A-CYCLIND-T2A-EGFP, or LXSH-TERT for the gene introduction. We introduced the packaging plasmids, pCMV-VSV-G-RSV-Rev, pCL-gag-pol, retrovirus plasmid (total 6μg) into 293T with lipofection. The virus supernatant was recovered and filtered with 0.45mm disk filter (code 17598, Sartorius AG, Göttingen, Germany), and we carried out the concentration of recombinant viruses with centrifuge and precipitation under the existence of PEG6000. The concentrated virus pellets from 30ml of supernatants were resuspended with 1.5ml of culture medium, and the viruses were used for the infection to the target cell with the existence of 4μg/ml of polybrene for 48 hours. The detailed procedure for the virus preparation as previously described [16].

**Fig 1:**
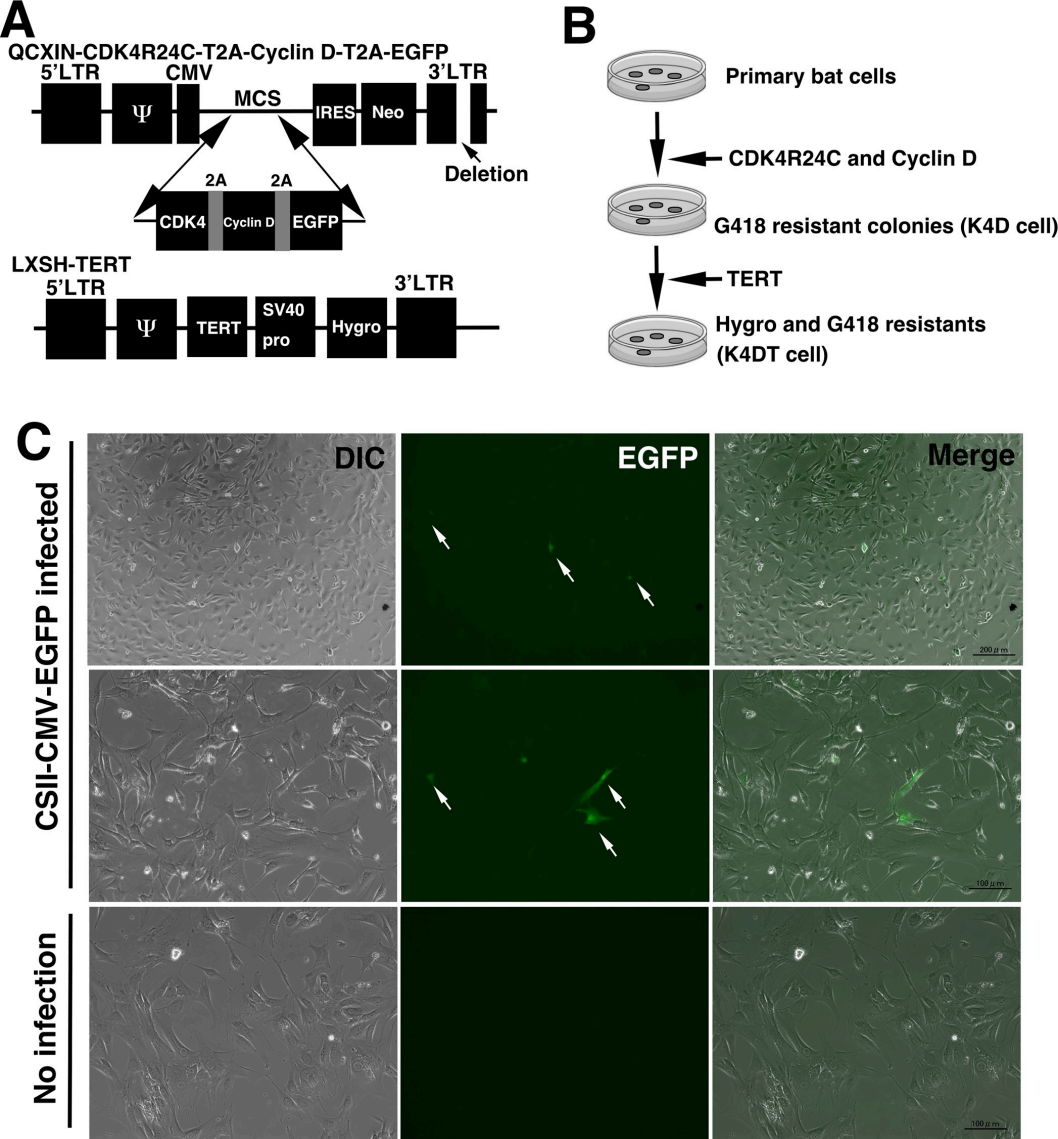
Schematic representation of establishment of cell and recombinant virus, which used in this study. A, Structure of recombinant MMLV-retroviruses used in this study. Upper panel, development of the poly-cistronic MMLV-retrovirus which expresses CDK4, CYCLIN D1, and enhanced green fluorescence protein (EGFP). Lower panel, Structure of MMLV-retrovirus that expresses telomerase reverse transcriptase (TERT), and Hygromycin resistant gene. B, Schematic representation for the establishment of immortalized cell lines from Bonin flying fox-derived primary cells. C, Detection of EGFP protein expression via lentivirus vectors in Bonin flying fox-derived cells. White arrows indicate the positive cells for EGFP expression. Left panels, cell morphology of differential interference contrast (DIC). Middle panels, expression of fluorescence protein detected by fluorescence microscopy. Right panels, merged images of DIC and fluorescence.

We chemically synthesized the protein-coding regions of cyclin dependent kinase 4 (*CDK4*) with R24C mutation, *CYCLIN D1*, and *EGFP* with Kozak sequence and restriction enzyme sites. We optimized the codon usage of cDNA fragment into the human. The cDNA inserts were ligated into the multiple cloning site of pQCXIN vector with restriction enzyme digestion, followed by ligation reaction. Fig 1A shows the structure of the recombinant MMLV-derived retrovirus used in this study. Based on the 1mg/ml of G418 antibiotic selection, the resistant recombinant cells were selected. Subsequently, we introduced the MMLV-retrovirus expressing telomere reverse transcriptase (TERT, pCLXSH-TERT). The infected cells were selected with G418 and hygromycin, to ensure the expression of *CDK4R24C* mutant, *CYCLIN D1*, and *TERT*. According to these sequential introductions, we named the cells as wild type, K4D (mutant CDK4, CYCLIN D1-expressing cells), K4DT (mutant CDK4, CYCLIN D1, and TERT-expressing cells).

### Western blot analysis and detection of telomerase activity with stretch PCR assay

We described the detailed methods for western blot and stretch PCR assay in our previous publications [17]. Briefly, primary antibodies used with their respective dilutions in this study are listed as follows: anti- CYCLIN D1 (1:5000, code no. 553, MBL, Nagoya, Japan), anti-CDK4 (1:2500, code no. K0065-3, MBL), and anti-alpha-tubulin (1:1000, cat. no. sc-32293, Santa Cruz Biotechnology, Dallas, TX, USA). Secondary antibodies used in this study are: sheep anti-mouse IgG linked horseradish peroxidase (HRP) (1:2000, code no. NA931 V, GE Healthcare, Little Chalfont, UK) and a donkey anti-rabbit IgG linked HRP (1:2000, code no. NA934 V, GE Healthcare). Enzymatic activity of the telomere elongation was detected using the commercially distributed kit ‘TeloChaser’ (code no. TLK-101, TOYOBO, Osaka, Japan). The detection procedure was executed by following a protocol provided by the manufacturer.

### Cell cycle analysis

We analyzed the cell cycle stage with Muse Cell Analyzer (Millipore, Burlington, Massachusetts, USA). We detected the cell cycle stage of wild type, cells expressing mutant CDK4, CYCLIN D1, and TERT. We used the staining kit from Millipore.

### Calculation of population doubling (PD)

From the end characters of introduced genes, we named the cell type. For instance, we termed the cells that express mutant CDK4 and CYCLIN D1 as “K4D” cells. Similarly, in case of cells expressing mutant CDK4, CYCLIN D1, and TERT, “K4DT” was assigned as the cell name. We did the calculation of population doubling of the wild type, K4D, and K4DT cells of Bonin flying fox. We carried out sequential passages to evaluate the growth of wild type and recombinant cells. We also assessed the PD value, which represents the number of cell divisions, calculated using the following formula; PD = log_2_ (a/b), where “a” is the number of cells counted at each passage and “b” is the number of cells seeded at the start of each passage [18,19]. To obtain the value of “a”, cells were seeded at a concentration of 5×10^4^ cells/well (the value of “b”) in a 6-well plate. When one of the cell types in the wells reached confluence, all the cells were trypsinized. After the trypsinization, we counted the number of cells in dishes (the value of "a") at the same time. We presented the results as the mean with standard deviation, from the experiments performed in triplicates.

### Senescence associated β-galactosidase (SA-β-Gal) staining

We used SA-β-Gal staining for the detection of cellular senescence. The staining was carried out with the cells at passage number 7 using the senescence detection kit (cat. No. K320-250, BioVision, Milpitas, California, USA) in the manufacturer's protocol.

### Chromosome analysis

We evaluated the G-banding pattern of fifty cells of wild type and K4DT for the determination of chromosome number. In brief, cells were treated with 0.02 μg/μl colcemid overnight, to increase the number of cells in the metaphase stage. We did the trypsinization of the cells, then we treated the cells with a hypotonic solution of 0.075 M potassium chloride and fixed using Carnoy’s fixative. Post-fixation, the cells were stained with Giemsa solution. We analyzed the detailed chromosomal patterns of 20 mitotic cells with G-banding method.

### F-actin staining

We carried out the cell culture of wild type and K4DT Bonin flying fox cells in the Falcon culture slide 354114, four well type (Corning, NY, USA) after the collagen treatment. Around 1.2 X 10^5^ cells of wild type and immortalized cells were seeded into each well. After the cells reached into sub-confluent condition, the cells were washed by 1 X PBS, then fixed with 4% paraformaldehyde solution for 5min. The fixed cells were permeabilized with 0.1% Triton X-100 solution in PBS for 10min. We stained the cells with X40 diluted Rhodamine X conjugated Phalloidin solution (Wako chemicals, 165–21641) in dimethylformamide (6.6μmols/L) and counterstaining (DAPI solution, Wako chemicals, 340–07971) for 20min. After the wash with 1X PBS, we obtained the fluorescence images with the X810 microscope (Keyence, Tokyo, Japan) with vectashield mounting medium (Maravai LifeSciences, San Diego, CA, USA).

### Interspecies somatic cell nuclear transfer (iSCNT)

Cumulus–oocyte complexes (COCs) were aspirated from 3- to 6-mm follicles of slaughtered bovine ovaries and were stored overnight at 10°C in saline [20]. After several washes with TCM199 (Thermo Fisher, Waltham, MA, USA) supplemented with 0.1% of polyvinylpyrrolidone (PVP), COCs were cultured in 4-well dishes (Thermo Fisher) in 500 μL of TCM199 supplemented with 10% of FBS (Thermo Fisher) covered with mineral oil, for 20 to 24 h at 39°C in a humidified atmosphere containing 5% of CO2. The COCs were denuded of cumulus cells by repeated pipetting in 0.1% hyaluronidase until the cumulus cells were removed completely. Oocytes with the first polar body were used in the experiments as MII oocytes. SCNT was carried out by a method reported previously [21]. For the preparation of the recipient ooplasm, maternal chromosomes were mechanically removed, and the removal was confirmed by DNA staining with 5 μg/mL Hoechst 33342 and visualization under UV light. A single immortalized Bonin flying fox derived cell or bovine granulosa cell was electrically fused with an enucleated oocyte and activated by applying two direct current (DC) pulses of 150 V/mm for 25 μsec with a 0.1-sec interval in 0.3 M mannitol containing 0.05 mM Ca^2+^ and 0.1 mM Mg^2+^, and then cultured in the manipulation medium containing 10 μg/mL cycloheximide (CHX) for 6 hours. After the activation, iSCNT embryos were cultured in KSOMaa supplemented with 5% of Fetal bovine serum in an atmosphere containing 5% of CO_2_, 5% of O_2_, and 90% of N_2_ at 39°C for 8 days. Each experiment was repeated more than three times, and the development to eight-cell on days 2 (day 0 was the day of nuclear transfer).

### PCR diagnosis

We used the following primers for amplification and diagnosis. Expression cassette of mutant CDK4-2A-CYCLIN D, product size is 350bp, TF955, 5'-GCTGGAGATGCTCACCTTCAA-3', TF956, 5'-TCCAGGTGGCCACGATCTTTC-3', Bat specific endogenous Tuberous sclerosis type II gene, product size is 498bp, TF959, 5'-CAGACCCTGCAGGACATTCTTG-3', TF960, 5'-AGTGTCCAGGAACTCCAGCAA-3', expression cassette of TERT, product size is 500bp, TF961, 5'-CTGCTCCTGCGTTTGGTGGATGATT-3', TF962, 5'-GTCCTGAGTGACCCCAGGAGTGGCA-3', expression cassette for EGFP, product size is 520bp, GFPF, 5’ -GGTCCACCTCTTCTTCTTCTTC-3’, GFPR, 5’ -CATCTACACCGACAACTCCATC-3’, Bovine oocyte derived mitochondria amplification (231bp), the forward primer bov Cytochrome oxidase subunit I F, 5’-CATCAACTTCATTACAACAATTATCAACATAAAG-3’) and reverse primer bov Cytochrome oxidase subunit I R, 5’- CCGAATGGTTCTTTTTTYCCTGAGTAGTA -3’) were used. All PCR reaction was carried out based on the provided from the manufacture of PCR enzyme, KOD-FX Neo (TOYOBO). We detected the PCR products in 1% agarose gel electrophoresis with Ethidium Bromide.

### Ethics statement

Dr. Hajime Suzuki found male Bonin flying fox with the injured condition at the barbed wire of central mountain area of Chichijima in Japan on January 20th in 2015. To prevent the poaching of the protected animals, we cannot disclose the detailed information for the location. Due to the damage of the right-wing and finger of found animal, Dr. Hajime Suzuki (Institute of Boninology, Chichijima, Ogasawara) brought the corresponding male to the emergency medicine area of Institute of Boninology, Chichijima. Dr. Hajime Suzuki and stuff of Institute of Boninology reported the finding of the injured Bonin flying fox to the protection office of Ministry of the Environment. The staff of the Institute of Staff of Boninology, including Dr. Hajime Suzuki, were trying to keep the health condition of male Bonin flying fox. However, the tissue of the damaged area of right-wing showed the necrosis and caused repeated breeding. Based on the clinical condition, the male Bonin flying fox was transferred to the Animal hospital of Conservation & Animal Welfare Trust, Okinawa on June 21st in 2016. On the June 22^nd^ in 2016, Dr. Takashi Nagamine and Dr. Yumiko Nakaya (Conservation & Animal Welfare Trust, Okinawa) carried out the surgery of the removal of 5^th^ finger of the right-wing and damaged wing tissue under the Isoflurane anesthesia. Furthermore, the health check of the injured male Bonin flying fox, the blood sampling was carried out. The blood sample and skin and tissue of 5^th^ finger of the right-wing were transferred to Ecological Risk Assessment and Control Section, Center for Environmental Biology and Ecosystem, National Institute for Environmental Studies, Tsukuba, Japan. Based on the tissues from the surgery, the primary cell culture was carried out on the National Institute for Environmental Studies, Tsukuba, Japan. The corresponding male Bonin flying fox was sent back to Institute of Boninology, Chichijima, Ogasawara, and joined into Experimental breeding population of Bonin flying fox. All records for the protection activity and surgical procedure and transfer were reported to the Ministry of the Environment.

## Results

### MMLV-retrovirus can efficiently introduce into Bonin flying fox cells, but a lentivirus does not

In our previous study, we introduced mutant *CDK4*, *CYCLIN D1*, and *TERT* as a mixture. We performed triple infection of monocistronic lentiviruses for immortalization [1,2,7,9]. At the first step, we exposed the EGFP-expressing lentivirus to the primary Bonin flying fox-derived cells. Surprisingly, even after 48 hours of infection, the limited number of cell population showed EGFP expression, indicating that lentivirus is not an efficient method for introducing the exogenous genes (Fig 1C). As the supportive evidence of successful packaging of lentivirus, we detected reasonable infection efficiency with the same lot of EGFP to rabbit derived muscle fibroblasts (S1 Fig). From these data, we decided to change the gene delivery method.

We next selected MMLV-retrovirus with VSV-G envelope. Interestingly, EGFP-expressing MMLV-retrovirus with VSV-G envelope protein showed around 10–20% efficiency for gene introduction. In the case of triple infection of monocistronic recombinant viruses, infection efficiency around 10–20% is not sufficiently high. We need the multiple infections of mutant *CDK4*, *CYCLIN D1*, and *TERT* for the immortalization. For this, we designed the polycistronic MMLV-retrovirus, which expresses both of mutant CDK4 and CYCLIN D1. As shown in Fig 1A, a mutant CDK4, CYCLIN D1, and enhanced green fluorescence protein (EGFP) protein would be expressed in polycistronic way. The internal ribosomal entry site *(IRES)-Neo* resistant gene exist at downstream of the *CDK4*, *CYCLIN D1*, and *EGFP*. We can select the recombinant cells with 1 mg/ml of G418 in the cultured medium. G418 selection ensured the introduction of mutant CDK4 and CYCLIN D1, and EGFP in Bonin flying fox cells, and we named the cells as “K4D cells” (mutant CDK4 and CYCLIN D1 introduced cells). We confirmed that wild type Bonin flying fox-derived cells could not survive in the presence of 1 mg/ml of G418. Consequently, after the G418 selection, we observed almost of the resistant cells showed the expression of EGFP, as expected from the structure of the expression system. We exposed MMLV-retrovirus, which expresses telomerase reverse transcriptase (*TERT*), and hygromycin resistant gene (Fig 1B) as the next step. We selected the double infected cells with hygromycin and G418 in the cultured medium, which ensured the introduction of both expression cassettes (mutant *CDK4-CYCLIN D1-EGFP* cassette, and *TERT* cassette). The established cells, harboring mutant CDK4, CYCLIN D, and TERT were named as “K4DT cells” (mutant CDK4, CYCLIN D1, and TERT expressing cells, Fig 1B).

### Detection of introduced protein with fluorescence and genomic PCR

**Fig 2:**
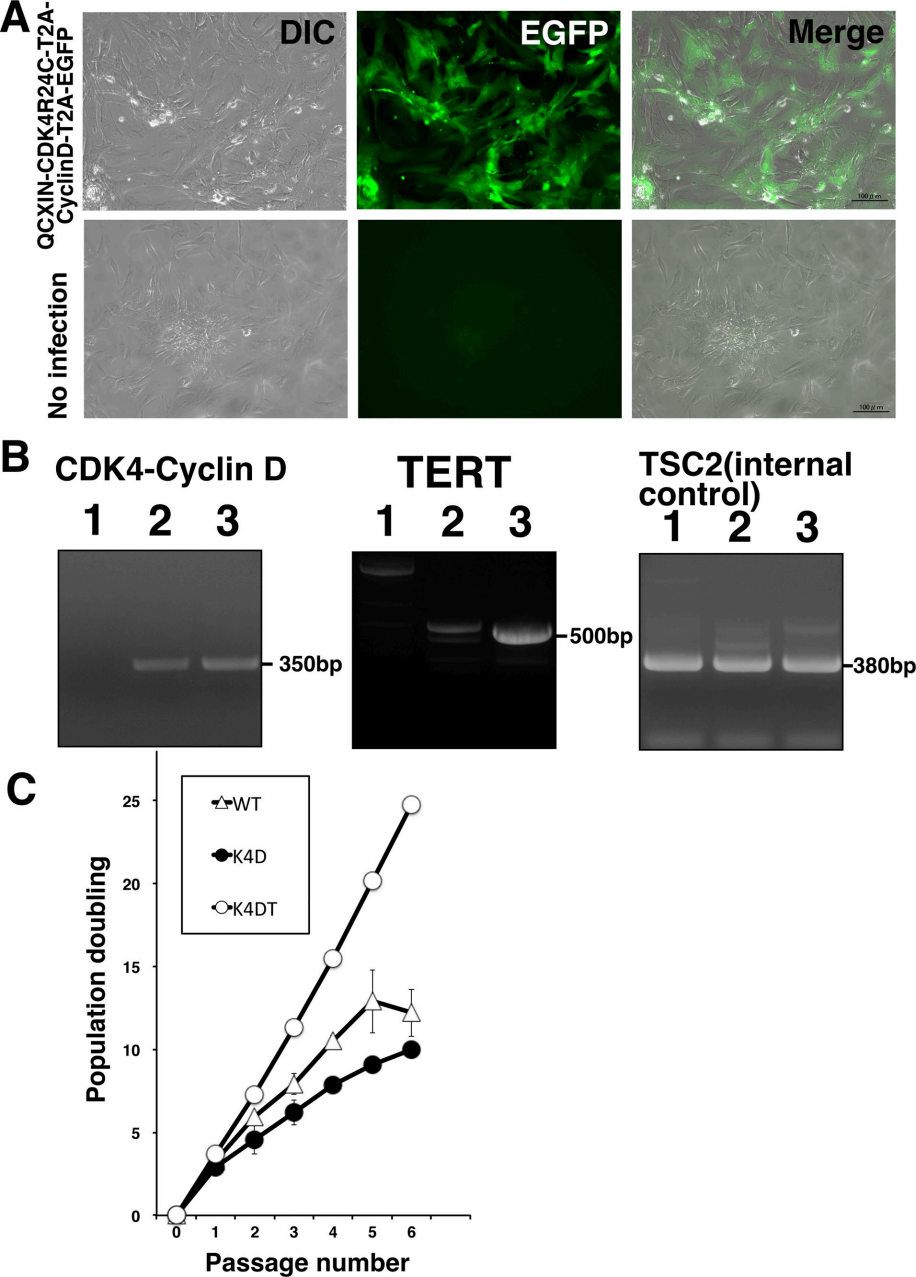
Detection of genomic integration and expression of introduced genes in Bonin flying fox-derived cells. A, Detection of fluorescence protein in Bonin flying fox-derived cells. Left panel, cell morphology in differential interference contrast (DIC). Middle panel, expression of fluorescence protein detected by fluorescence microscopy. Right panels, merged images of DIC and fluorescence. B, Detection of genomic integration of expression cassette by polymerase chain reaction (PCR). PCR amplification with expression cassette for CDK4-CYCLIN D1 (left panel), expression cassette for TERT (middle panel), internal control gene (Right panel, bat-derived endogenous TSC2 gene; Tuberous Sclerosis Type II). 1, wild type cell; 2, K4D cell; 3, K4DT cell. C, Cell growth curve under the sequential passages of wild type, K4D, and K4DT cells. Average cell number and standard error were calculated from triplicated samples.

To ensure the introduction of expression cassette of mutant CDK4 and CYCLIN D1, we carried out the detection of protein expression in wild type, K4D, and K4DT cells using fluorescence. As shown in Fig 2A, the MMLV-retrovirus which carries mutant CDK4 and CYCLIN D1, and EGFP protein showed a reasonable expression level of EGFP protein in Bonin flying fox-derived cells. Furthermore, the results of genomic PCR showed that the expression cassettes of *CDK4-CYCLIN D1* and *TERT* were successfully inserted into the Bonin flying fox-derived cells (Fig 2B and S2 Fig). In addition to the PCR analysis, we carried out the sequential passages of wild type, K4D, and K4DT cells, as shown in Fig 2C. Although the wild type and K4D cell could not continue cell proliferation, K4DT cells showed cell proliferation more than 25 of population doubling (PD) value. Based on these observations, we concluded that we successfully introduced the expression cassette of mutant *CDK4-CYCLIN D1*, and *TERT* into Bonin flying fox-derived cells (Fig 2C).

### Detection of introduced genes with western blotting and enzymatic activity of telomerase

**Fig 3:**
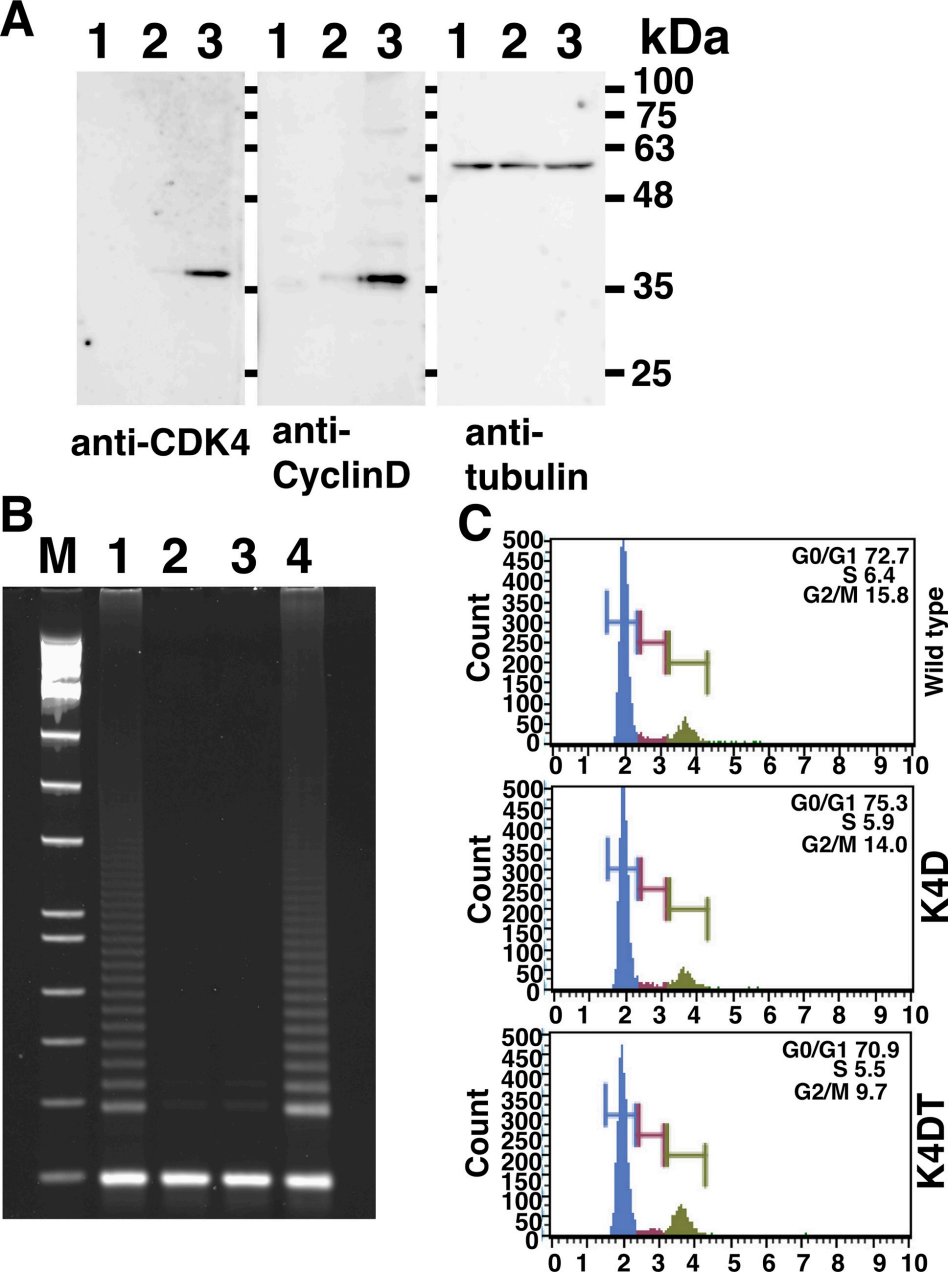
Detection of protein expression of introduced genes and the enzymatic activity of the telomere reverse transcriptase. A, Left panel, western blotting with CDK4 antibody, Middle panel, western blotting with CYCLIN D1 antibody, western blotting with anti-tubulin. 1, wild type cell; 2, K4D cell; 3, K4DT cell. B, Detection of enzymatic activity of TERT with stretch PCR assay. M, Molecular weight marker, 1, Hela cell extract; 2, wild type cell extract; 3, K4D cell extract; 4, K4DT cell extract. C, Cell cycle analysis of established cells, Upper panel, wild type cell; Middle panel, K4D cell; Lower panel, K4DT cell.

We used the total proteins from wild type, K4D, and K4DT Bonin flying fox-derived cells for western blotting. As shown in Fig 3A and S3 Fig, the anti-CDK4 and anti-CYCLIN D1 antibodies efficiently detected the expression of the introduced expression cassette. Interestingly, the protein expression level of K4D cells (Fig 3A, lane 2) was much lower than that of K4DT cells (Fig 3A, lane 3). This result suggests that the expression of TERT enhances the expression of mutant CDK4 and CYCLIN D1. We detected the enzymatic activity of telomerase with stretch PCR. As shown in Fig 3B and S4 Fig, the positive control, HeLa cell extract, and Bonin flying fox-derived K4DT cells showed enzymatic activity to extend the telomere sequence. From these data, we concluded that our established K4DT cells possess an enzymatic activity to extend the telomeric repeat.

### K4DT cells of Bonin flying fox-derived cells are free from cellular senescence

**Fig 4:**
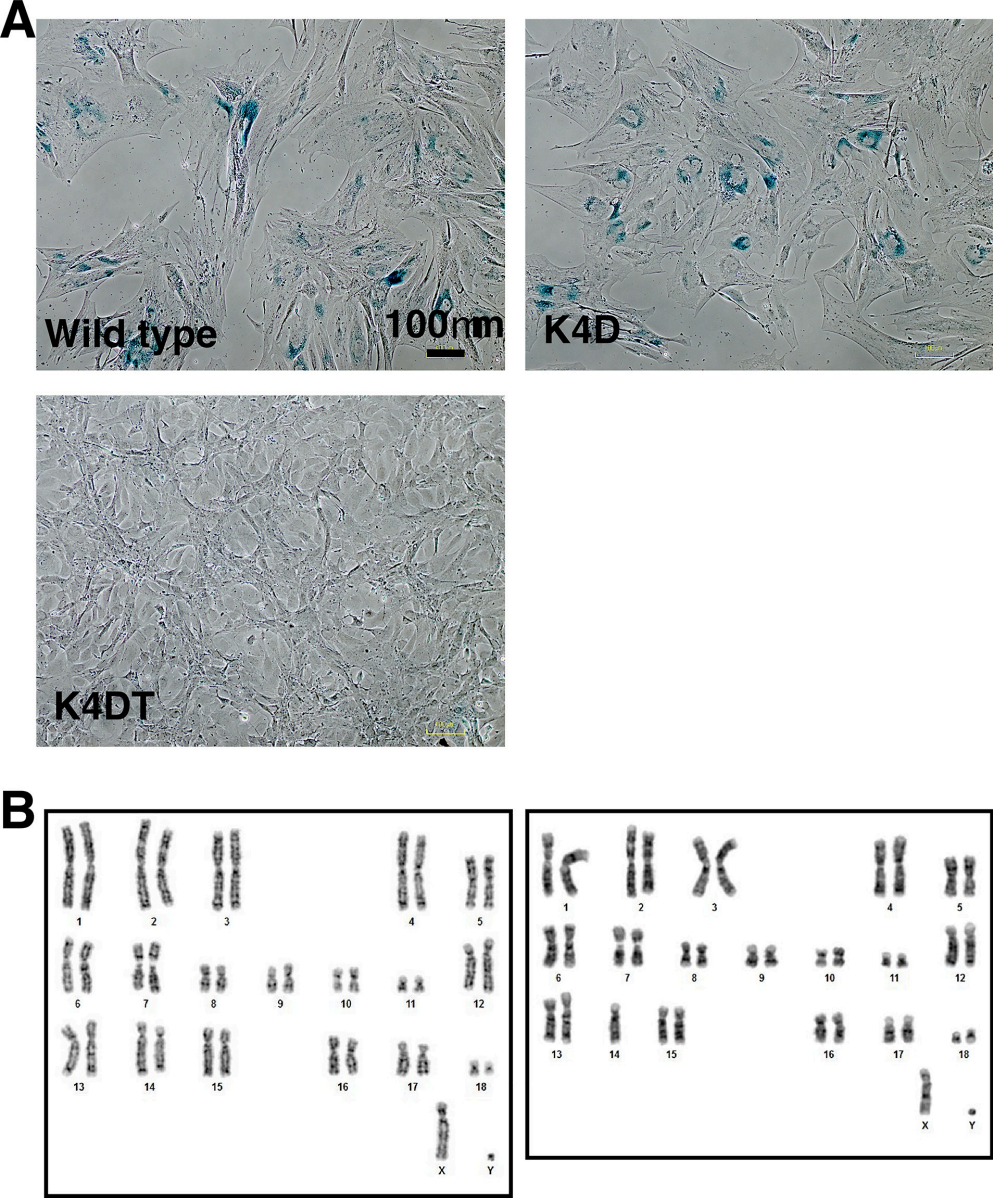
Detection of cellular senescence of established cells and chromosome analysis. A, Results of senescence-associated beta-galactosidase staining. The results of wild type, K4D, and K4DT cells are shown. The positive staining parts have been highlighted by blue deposit. Note that K4DT cells are free from blue staining in the cytoplasm. B, Chromosome analysis of the wild type and immortalized Bonin flying fox with G-banding method. Left panel, chromosome pattern of wild type Bonin flying fox; Right panel, chromosome analysis of K4DT cell from Bonin flying fox. It is to be noted that one of the chromosome 14 is missing.

As shown in Fig 4A, the wild type cells at passage 7 showed enlarged cytoplasm, a characteristic of senescence-associated cellular phenotype. Interestingly, K4D cell also showed larger cytoplasm, while the K4DT cells retained a smaller cell size at passage 7 (Fig 4A, lower panel). Furthermore, although the wild type and K4D cells were positive for senescence-associated β-galactosidase (SA-β-gal) staining, K4DT cells were negative (Fig 4A). From these results, we concluded that our established Bonin flying fox-derived K4DT cells are free from the cellular senescence.

### Karyotype and cell cycle analysis of wild type, K4D, and K4DT

Since the pattern of G-banding of Bonin flying fox has not been reported yet, we first analyzed the normal chromosomal pattern with primary cells. As shown in the left panel of Fig 4B, the wild type cell showed 2n = 38 + XY chromosomes with a unique banding pattern. Our data is the first result describing the details of Bonin flying fox. Secondly, we analyzed the karyotype of Bonin flying fox-derived K4DT cells. Although we observed the monosomy of chromosome 14, there was no abnormality in the rest of the chromosomes. Furthermore, we analyzed the cell cycle stages of the wild type and K4DT Bonin flying fox-derived cells. We showed the representative results of the detection in Fig 3C. We did not observe any difference in the ratio of G1/S and G2/M ratios between wild type and K4DT cells (Table 1). This data indicates that K4DT cell maintains original conditions for the turnover rate of the cell cycle. From these data, we conclude that we successfully established the immortalized cell lines from the critically endangered megabat, Bonin flying fox.

**Table 1:** Summary of the karyotyping of wild type and established K4DT cell.

Number of cells\ Chromosome number	37	38	39
**wild type cell**	**0/50**	**50/50**	**0/50**
**K4DT cell**	**45/50**	**5/50**	**0/50**

The number of mitotic cells, which showed the specific number was shown in the table. We analyzed 50 mitotic bodies for each cell line.

### F-actin distribution of wild type and K4DT Bonin flying fox derived cells

**Fig 5:**
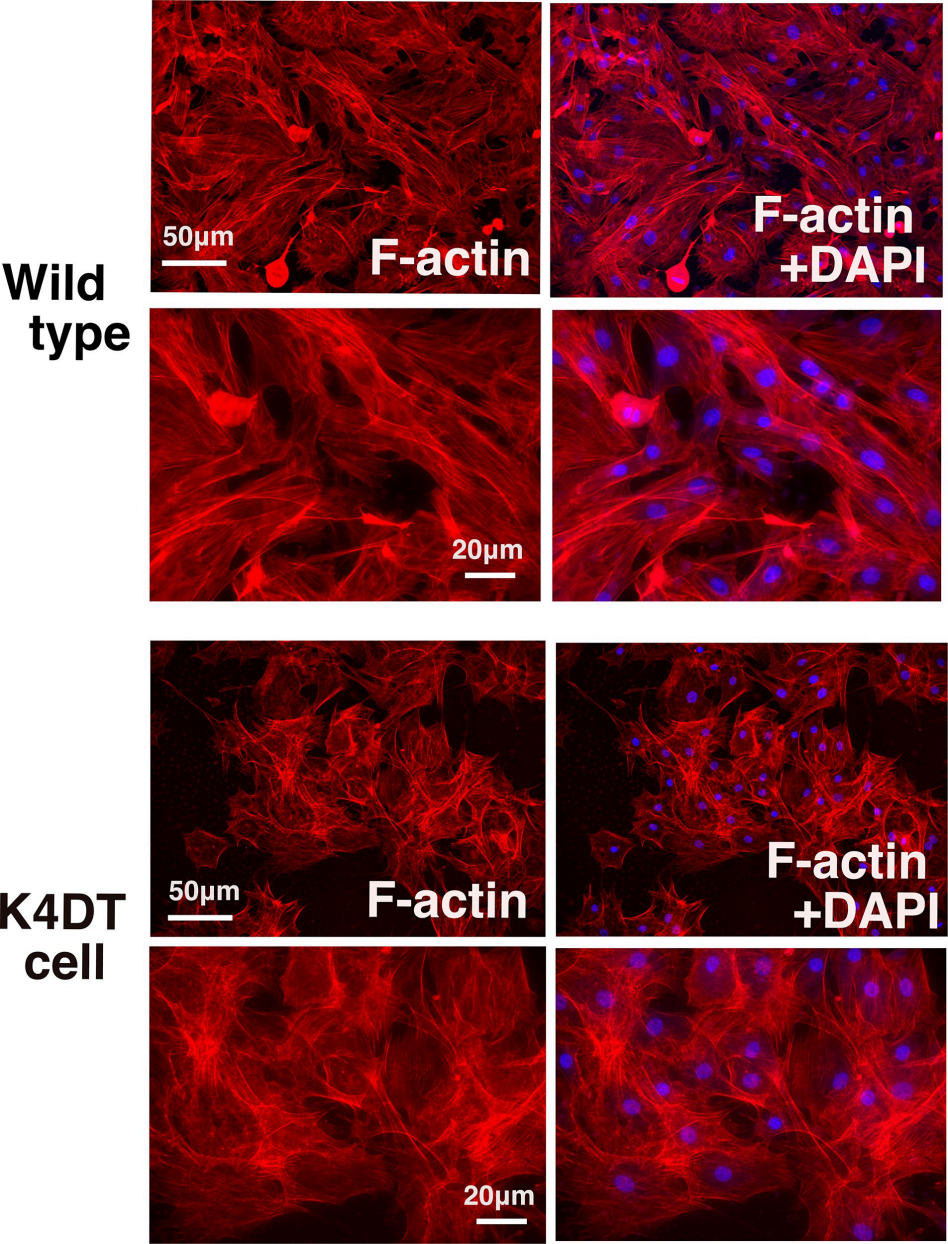
Detection of cytoskeletal F-actin and nuclear staining of wild type and K4DT cell. Upper four panels. Low and high magnification of wild type Bonin flying fox derived cell stained with F-actin and nuclear staining (DAPI). Low and high magnification of K4DT cell derived from Bonin flying fox. Note that we cannot see the difference of F-actin distribution and nuclear morphology between wild type and K4DT cells.

Although we detected the monosomy of chromosome 14, we evaluated if our established K4DT cell keeps original nature as so far. As shown in Fig 5, we stained F-actin, which is a major factor to control the cell migration and movement. As the results of the staining, we could not observe a significant difference between wild type, and K4DT cell of Bonin flying fox derived cells.

### Interspecies somatic cell nuclear transfer (iSCNT) of the immortalized cell into bovine embryo showed development to 8 cell stage

**Fig 6:**
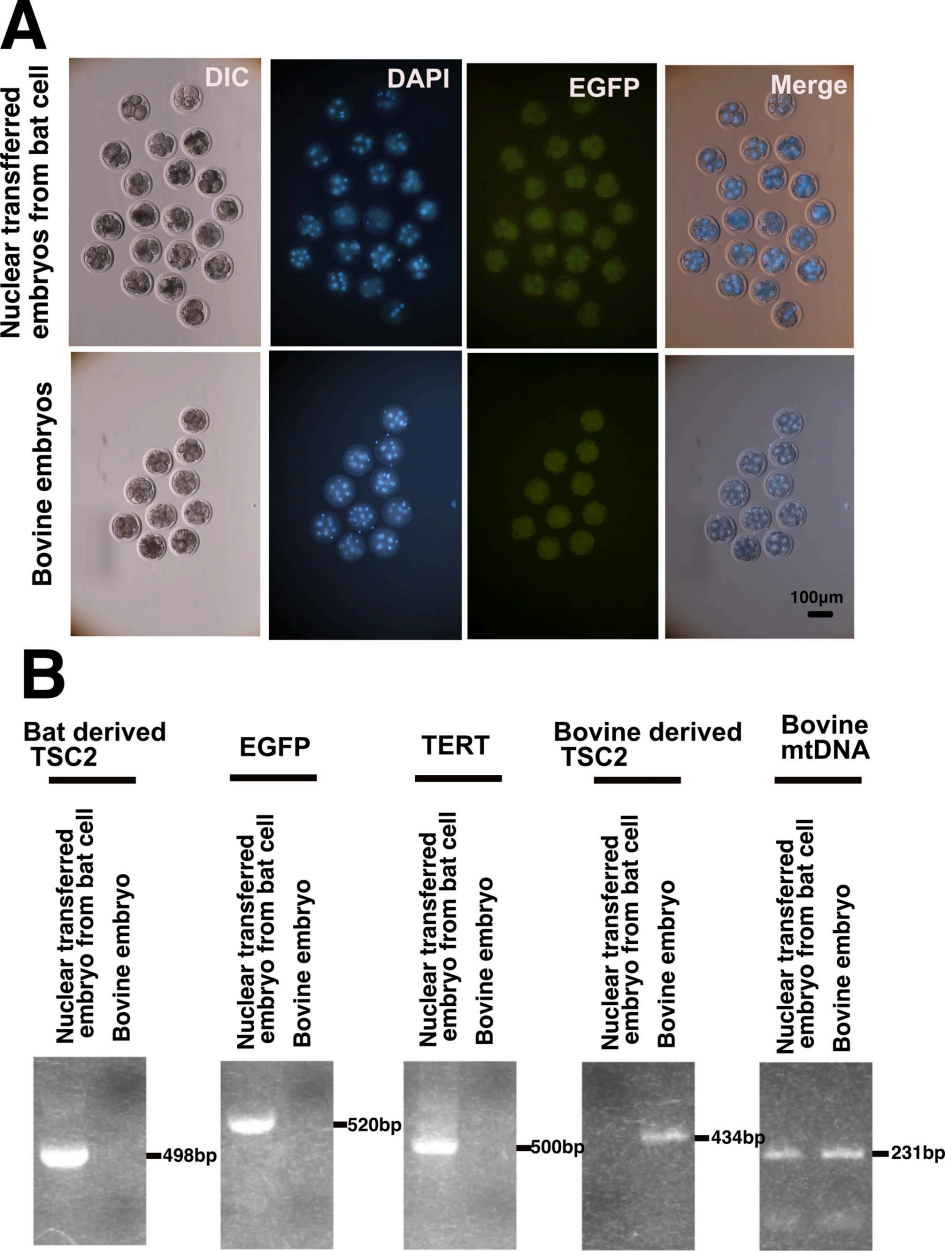
Interspecies somatic cell nuclear transfer (iSCNT) of Bonin flying fox derived cell into the bovine-derived embryo. A, Morphology of nuclear-transferred embryo and bovine-derived wild type embryos after 48 hours. Note that DAPI staining showed that embryo contains multiple nuclei around 8 cell stages. EGFP panels showed fluorescence derived from EGFP expression is not detectable. B, Genomic PCR analysis of nuclear-transferred embryo from bat cell and bovine wild type embryo. Tuberous sclerosis type II (TSC2) gene was used for specific primers for bat derived genome (panel of bat derived TSC2) and bovine genome (panel of bovine-derived TSC2). The amplification product was observed only in the nuclear-transferred embryo from bat cell, with bat derived TSC2 primers, EGFP primers, TERT primers. Bovine TSC2 primers showed amplification only from the bovine embryo (Panel of bovine-derived TSC2). Furthermore, the amplification products were commonly observed with specific primers with bovine mitochondrial DNA (panel of Bobine mt DNA).

To address the potential usefulness of the immortalized cell from Bonin flying fox, we carried out the interspecies somatic cell nuclear transfer (iSCNT) from immortalized Bonin flying fox cell to the bovine oocyte. After the 48 hours, the iSCNT embryo reached at 8 cell stages, as shown in Fig 6A. In the EGFP panel of Fig 6A, the embryo did not show the expression of EGFP, which possibly explained by the genomic reprogramming process after the somatic nuclear transfer. However, the DAPI staining clearly showed that iSCNT technology allowed the embryonic development and successful cell division from oocyte to 8 cell stages (Fig 6A, the panel of DAPI). To biologically detect whether the d our iSCNT was successful in the embryos, we carried out PCR amplification with bat specific PCR primers, as shown in Fig 6B and S5 Fig The iSCNT embryo showed amplification of DNA fragment with bat genome-specific primers. However, wild type bovine embryo-derived genome did not (Bat derived TSC2 panel). The results from amplification EGFP (Fig 6B, EGFP panel) and TERT (Fig 6B, TERT panel) specific primers showed consistent results with that from bat derived TSC2. Interestingly, PCR amplification with bovine-derived TSC2 primers, which are specific to the bovine genome, showed amplification of DNA fragment from the bovine embryo. However, bovine-derivedTSC2 primers did not show amplification of DNA fragment from iSCNT embryo (Fig 6B, bovine-derived TSC2 panel). These results showed that the genome of iSCNT embryo has all replaced by the successful nuclear transfer from immortalized Bonin flying fox derived cells. To verify if we succeeded in the recovery of DNA from both of embryo, we carried out the PCR amplification with PCR primers, which are specific to mitochondrial DNA of bovine. In the process of nuclear transfer, although the nuclear of the oocyte would be replaced, the mitochondrial DNA in the cytoplasm will not be replaced, and keep the original origin of the oocyte. In this study, we carried out the PCR amplification with bovine mitochondrial DNA. As shown in Fig 6B (Bovine mt DNA penal), iSCNT embryo, and wild type bovine embryo, both of the sample showed the amplification of DNA fragment of mt DNA. From these data, we concluded that iSCNT procedure was successful. We concluded that nuclei of the immortalized Bonin flying fox derived cells keeps original functionality for cell division within early development, although monosomy of chromosome 14 was observed around 90% of cells.

## Discussion

We previously showed that the expressions of mutant CDK4 and CYCLIN D1, and TERT allow us to efficiently establish the immortalized cells in various species, including human [8], bovine and pig [1,2], monkey [3], midget buffalo [4], prairie vole [5], sea turtle [9], leopard cat [7], and rat [22]. In human, bovine, pig, monkey, midget buffalo, and prairie vole, the wild cat-derived cells showed the cellular immortalization with the combination of mutant CDK4, CYCLIN D1, and TERT. However, it is interesting to note that the African elephant-derived cell showed cellular senescence even after the introduction of these three genes [23]. Furthermore, in the case of chick-derived cells, the efficiency of cellular immortalization becomes dramatically lower when compared with that of mammalian cells [24]. In this study, we successfully attained immortalized cells from Bonin flying fox-derived primary cells. Previously, there are two manuscripts which described the establishment of primary and immortalized cells from black flying fox and Pteroid bat. Our manuscript focusses on the Bonin flying fox, which is one of the critically endangered animals.

In the previous omega bat derived cells, TERT expression allowed the establishment of immortalized cell lines. However, we need to pay attention to the accumulation of p16 senescence protein. In the case of human-derived cells, Haga et al. showed that skin-derived cell could be immortalized only with TERT expression. However, the mammary gland epithelial cell and lung epithelial cells did not show the immortalization with TERT expression [25]. The mammary gland epithelial cell and lung epithelial cells required knockdown of p16 or expression of Bmi1 was required for the [25][25]immortalization. Additionally, they found out p16 senescence protein level has elevated in mammary gland epithelial cell and lung epithelial cells in the cell passage. However, the skin-derived cell did not show the accumulation of p16 protein. In this study, Bonin flying fox cell showed the enlarged cytoplasm, which is one of the major characteristics of senescence cells (Fig 4A). From the cellular morphology, Bonin flying fox derived cells showed enlarged cytoplasm. The enlarged cytoplasm suggests that the protein level of p16 senescence protein caused in the cell culture. From these supportive side evidence, inactivation of the p16-RB pathway would be needed for immortalization of Bonin flying fox derived cells.

As a traditional immortalization method, expression of oncogenic proteins, such as SV40 virus large T antigen or human papillomavirus (HPV)-derived E6E7 have been used [22,26]. However, SV40 and E6E7 proteins cause the inactivation of p53 tumor suppressor protein, which is highly critical to maintaining the accuracy of the genome. Due to the loss of function of p53, the methods as mentioned above frequently induce chromosome abnormality and genomic instability. We previously reported that the expression of SV40 large T causes tetraploid formation in around 20% of cells after 8 passages in pig embryonic fibroblasts [26]. In this study, the tetraploid formation was not evident despite of the monosomy of chromosome 14 observed in Bonin flying fox-derived cells. Expressions of mutant CDK4, CYCLIN D1, and TERT are more advantageous to keep the original nature of the cells.

In previous studies of Low land anoa and Tsushima leopard cat, K4D cells continued to proliferate even after the wild type cells stopped [4,7]. Interestingly, in case of Bonin flying fox bat, the K4D and wild type cells stopped cell proliferation almost at the same passage number. The cellular stress during the cell culture might explain the halt of cell proliferation of K4D cells during the sequential passages. Moreover, the expression levels of introduced genes might vary, depending upon the cell and tissue type.

We observed the partial instability of chromosome 14 in Bonin flying fox-derived immortalized cells. The chromosome stability is mainly regulated by the function of the tumor suppressor gene, p53. Immortalized cells derived from Bonin flying fox are responsible for maintenance of the wild type function of p53. However, as a result of chromosome analysis, detected monosomy of chromosome 14 probably indicated that the role of p53 might be partially lost in the Bonin flying fox-derived cells.

To evaluate the potential usefulness of immortalized cells for the regeneration of megabat individuals, we carried out Interspecies somatic cell nuclear transfer (iSCNT) of immortalized Bonin flying fox derived cells into bovine oocyte. We shoed that we successfully obtained 8 cell stage embryo from iSCNT. This manuscript is the first report which describes the successful embryo development of Megabat with iSCNT technique.

Although we succeeded in the establishment of immortalized cell population derived from Bonin flying fox, there is a high heterogeneity for target cells. As shown in Fig 2A, the expression level of EGFP varies from cell to cell. The immortalization of K4DT method is so efficient that these variations exist even in the mass population of immortalized cells. However, to detect the detailed characteristics of the immortalized cell, the cell cloning procedure with dilution would be necessary. As shown in S6 Fig, we are middle in the process of the pick up of the colonies. The comparison of the expression level of introduced mutant CDK4 and CYCLIN D1 might explain the biological difference among multiple clones.

Our study enabled us to establish immortalized cells from Bonin flying fox bat successfully and showed that immortalized cells might be useful for the re-generation of embryos. This work would contribute to the scientific significance of the cryopreservation of cells derived from endangered animals.

## Conclusions

In this study, we successfully established the immortalized cell from the primary cell of Bonin flying fox, which critically endangered animal with the expression of mutant CDK4, CYCLIN D, and telomere reverse transcriptase (TERT). The established cell showed the monosomy of chromosome 13; the rest of the chromosome condition was intact. After the interspecies somatic cell nuclear transfer (iSCNT), the nuclei of the Bonin flying fox derived immortalized cell developed until 8 cell stage. Our established cell has the potential to contribute to the regeneration of the critically endangered bat.

## Supporting information

S1 Fig Detection of infection efficiency of EGFP expressing lentivirus into rabbit derived muscle fibroblasts. Upper six panels, low and high magnification of rabbit muscle fibroblasts infected with CSII-CMV-EGFP. 48 hours after the stop of the infection. Lower three panels, rabbit derived muscle.
(TIFF)

S2 Fig No crop gel images of the PCR diagnosis of Fig 2B. The corresponding area of the gel images were indicated by white rectangles.
(TIFF)

S3 Fig No crop blot images of the western blots of Fig 3A. The corresponding area of the blots were indicated by black rectangles.
(TIFF)

S4 Fig No crop gel image of the stretch PCR of Fig 3B. (TIFF)

S5 Fig No crop gel image of PCR amplifications listed in Fig 6B. The corresponding area of the gel images were indicated by white rectangles.
(TIFF)

S6 Fig Immortalized Bonin flying fox derived cell, which spread under the diluted condition for isolation of clones. EGFP Fluorescence can be detected which come from the expression cassette.
(TIFF)
