# Correction: Establishment of immortalized primary cell from the critically endangered Bonin flying fox (*Pteropus pselaphon*)

**DOI:** 10.1371/journal.pone.0234054

**Published:** 2020-05-29

**Authors:** Tetsuya Tani, Takahiro Eitsuka, Masafumi Katayama, Takashi Nagamine, Yumiko Nakaya, Hajime Suzuki, Tohru Kiyono, Kiyotaka Nakagawa, Miho Inoue-Murayama, Manabu Onuma, Tomokazu Fukuda

After publication of this article [1], concerns were raised by a reader regarding (1) inaccuracies in population size, species distribution and International Union for the Conservation of Nature (IUCN) classification of the Bonin Flying Fox, (2) insufficient rationale for the study, (3) missing references, and (4) missing details about the ethics clearance for the study. Here, the authors provide additional information to clarify these issues.

The Bonin Flying Fox is incorrectly referred to as “critically endangered” throughout the article. Although the Bonin Flying Fox (*Pteropus pselaphon*) used to be classified as critically endangered, their current status is classified by the IUCN as “endangered” (EN). Presently the estimated number of Bonin flying foxes is around 300–400 individuals [2].

The authors have provided more contextualization for the following statement from the final sentence of the Introduction: “The establishment of Bonin Flying Fox-derived immortalized cells would contribute to understanding the diversity and branching out of the Bonin flying fox species from other is megabats during evolution.” The authors have provided the following comments:

“The Bonin Flying Fox is a Japanese endemic species and is recognized as a ‘Natural monument of Japan.’ This species is still at risk of extinction. As a biological specimen of this endemic species, the preservation of cultured cells is an important mission. Other countries, such the United States and United Kingdom, started preserving biological specimens of endangered animals in frozen zoos and frozen ark projects. Our research team started preserving the cells of endangered animals of original species of Japan as an analogous frozen zoo project. Furthermore, the preservation of immortalized cells of critically endangered species would enable us to share these cells among scientists worldwide, resulting in the progress of genetic studies on megabats.”

Three sentences in [1] have errors and missing references for the cited literature. The corrected sentences are provided below. The corrections do not change the overall conclusions. Please see the location of the error, the original text, the author-corrected text, and the corresponding references here.

**Table:** 

**Location**	**Original text**	**Corrected text**	**Corresponding references**
Introduction, second sentence	“During World War II, Bonin flying fox was hunted and exported as military food to Guam Island.”	“After World War II, Bonin Flying Fox was hunted and exported as food to Guam Island (Abe et al., 1994).”	Abe M, Maeda K, Ishii N, Sano H. Distribution and behavior and lifestyle of Ogasawara mega bat. Ogasawara Res (Ogasawara Kenkyu Nenpou) Japanese. 1994;18: 1–3
Introduction, fifth sentence	“However, in 1986, a colony of Bonin flying fox was found in a cave of Chichijima, one of the Bonin Islands.”	“However, in 1986, a colony of Bonin Flying Fox was found in Chichijima, one of the Bonin Islands (Abe et al., 1994).”	Abe M, Maeda K, Ishii N, Sano H. Distribution and behavior and lifestyle of Ogasawara mega bat. Ogasawara Res (Ogasawara Kenkyu Nenpou) Japanese. 1994;18: 1–3
Discussion, sixth sentence	“Previously, there are two manuscripts which described the establishment of primary and immortalized cells from black flying fox and Pteroid bat.”	“Previously, there are two manuscripts which described the establishment of primary and immortalized cells from Black Flying Fox [13] and Pteroid bat [14].”	Crameri G, Todd S, Grimley S, McEachern JA, Marsh GA, Smith C, et al. (2009) Establishment, Immortalisation and Characterisation of Pteropid Bat Cell Lines. PLoS ONE 4(12): e8266. https://doi.org/10.1371/journal.pone.0008266 Laing E, Sterling S, Weir D, Beauregard C, Smith I, et al. (2019) Enhanced Autophagy Contributes to Reduced Viral Infection in Black Flying Fox Cells. Viruses 11: 260. Available: http://dx.doi.org/10.3390/v11030260

Finally, the following text should be included as the second paragraph in the Ethics Statement section:

“The Institute of Boninology has official permission from the Government of Japan to handle species falling under all Japanese wildlife protection laws. The Institute of Boninology sent official records of the finding of an injured Bonin Flying Fox (Document number, IBO20160120) to (1) the Tokyo metropolitan Ogasawara Island branch office, section of bird and wild animals (Wildlife Protection law related, under the Ministry of Internal Affairs and Communications of Japan), (2) the Board of Education of Ogasawara Village (Cultural Property Protection Law related, under the Agency for Cultural Affairs and Ministry of Education, Culture, Sports, Science and Technology of Japan), and (3) the Nature Conservation Officer Office (Law for the Conservation of Endangered Species related, under the Ministry of the Environment of Japan).”
