# Supplementary figures and images for: Establishment of immortalized primary cell from the critically endangered Bonin flying fox (Pteropus pselaphon)
Source: PLoS One. 2019 Aug 26;14(8):e0221364. doi: 10.1371/journal.pone.0221364 (PMC6709887; doi:10.1371/journal.pone.0221364)

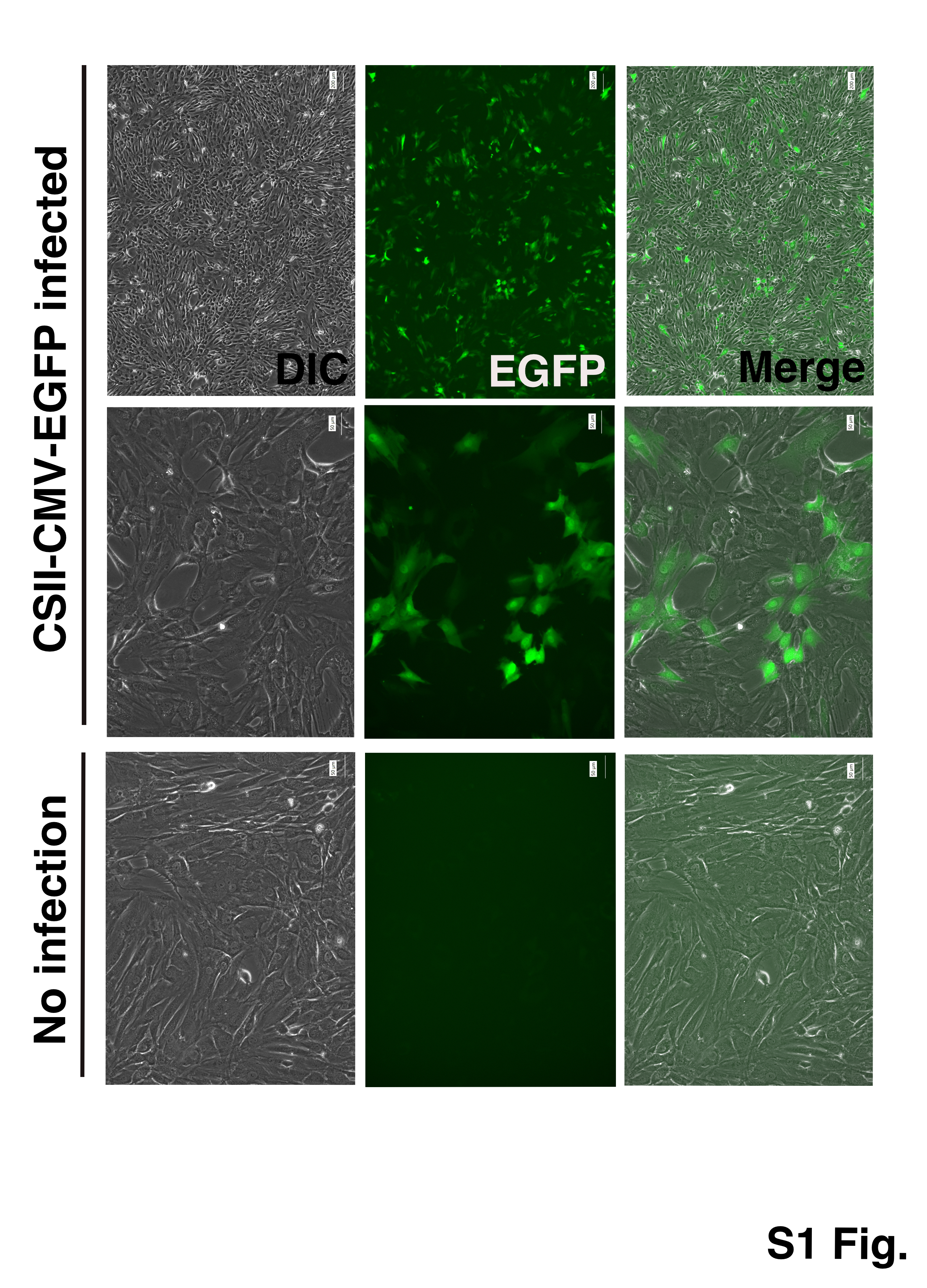

Supplement: S1 Fig — Upper six panels, low and high magnification of rabbit muscle fibroblasts infected with CSII-CMV-EGFP. 48 hours after the stop of the infection. Lower three panels, rabbit derived muscle. (TIFF) [file pone.0221364.s001.tiff]

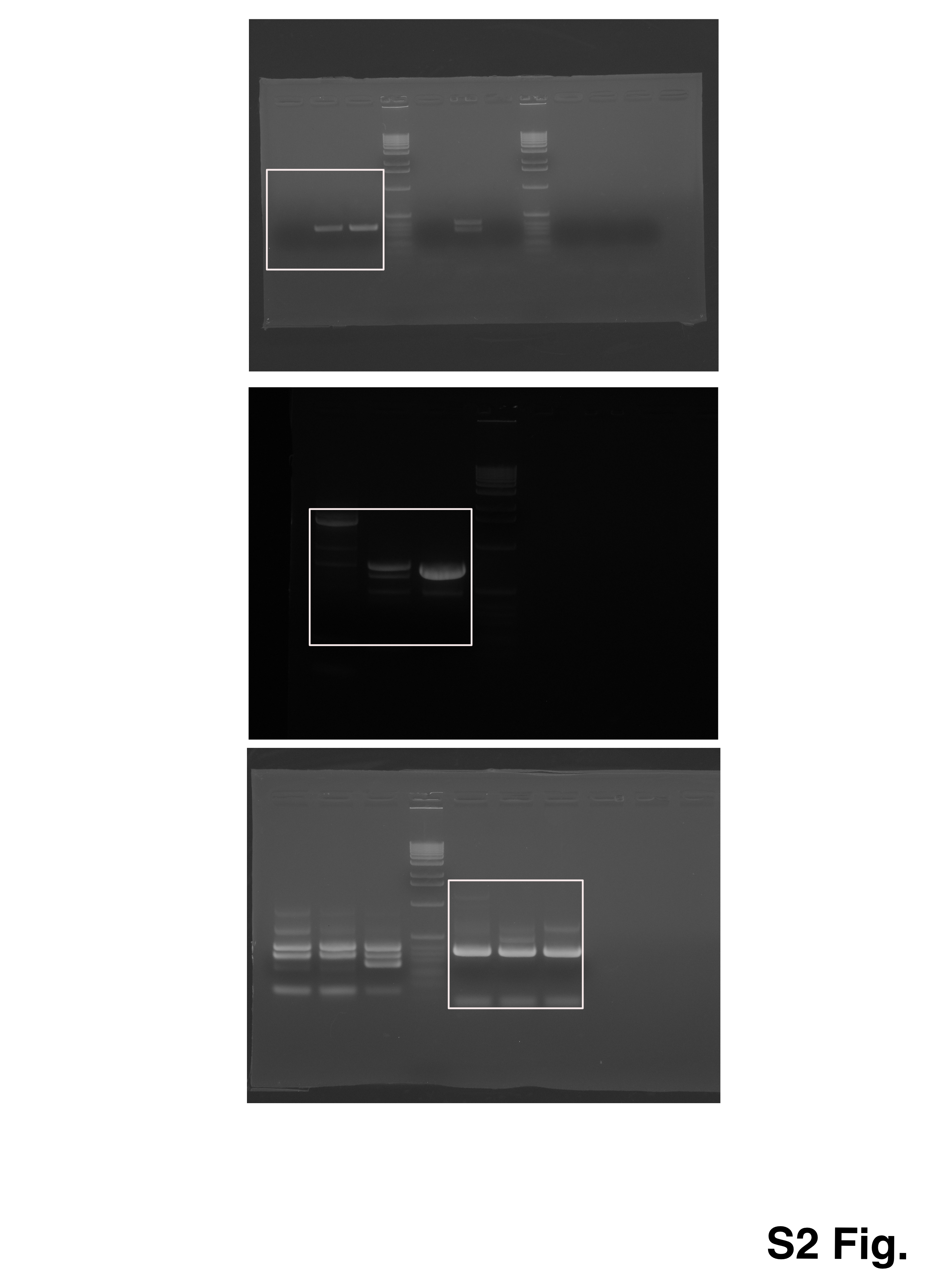

Supplement: S2 Fig — The corresponding area of the gel images were indicated by white rectangles. (TIFF) [file pone.0221364.s002.tiff]

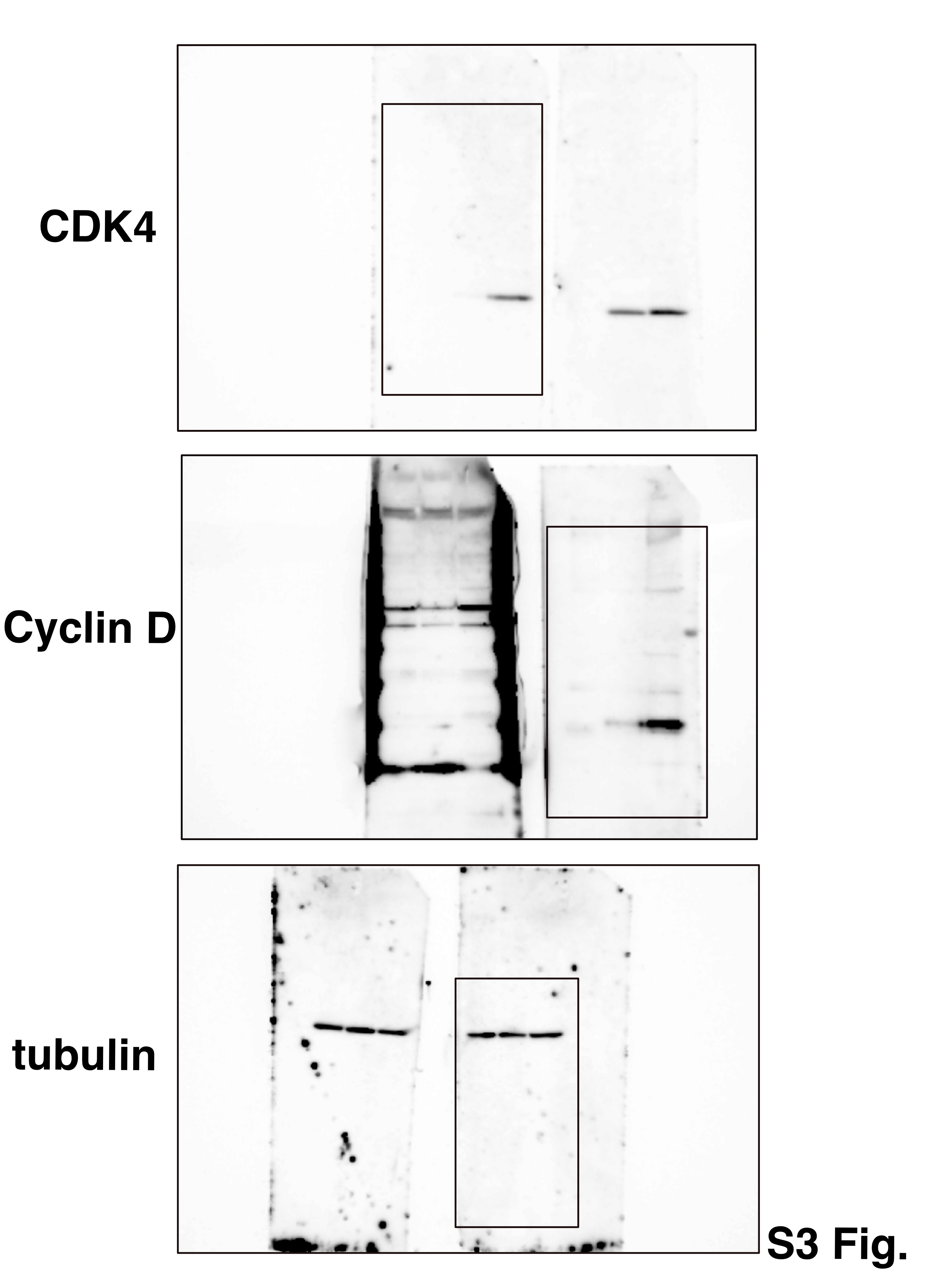

Supplement: S3 Fig — The corresponding area of the blots were indicated by black rectangles. (TIFF) [file pone.0221364.s003.tiff]

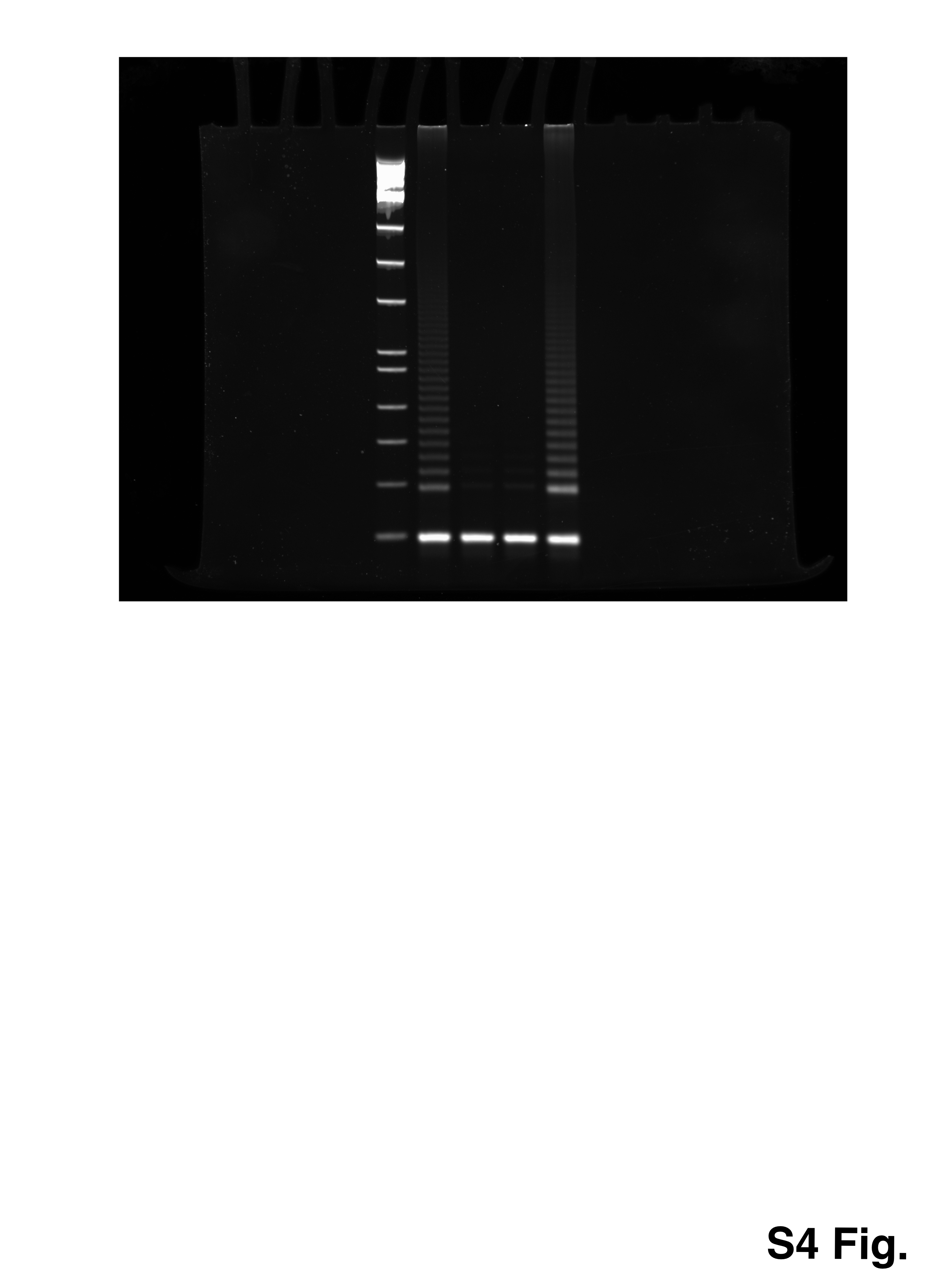

Supplement: S4 Fig — (TIFF) [file pone.0221364.s004.tiff]

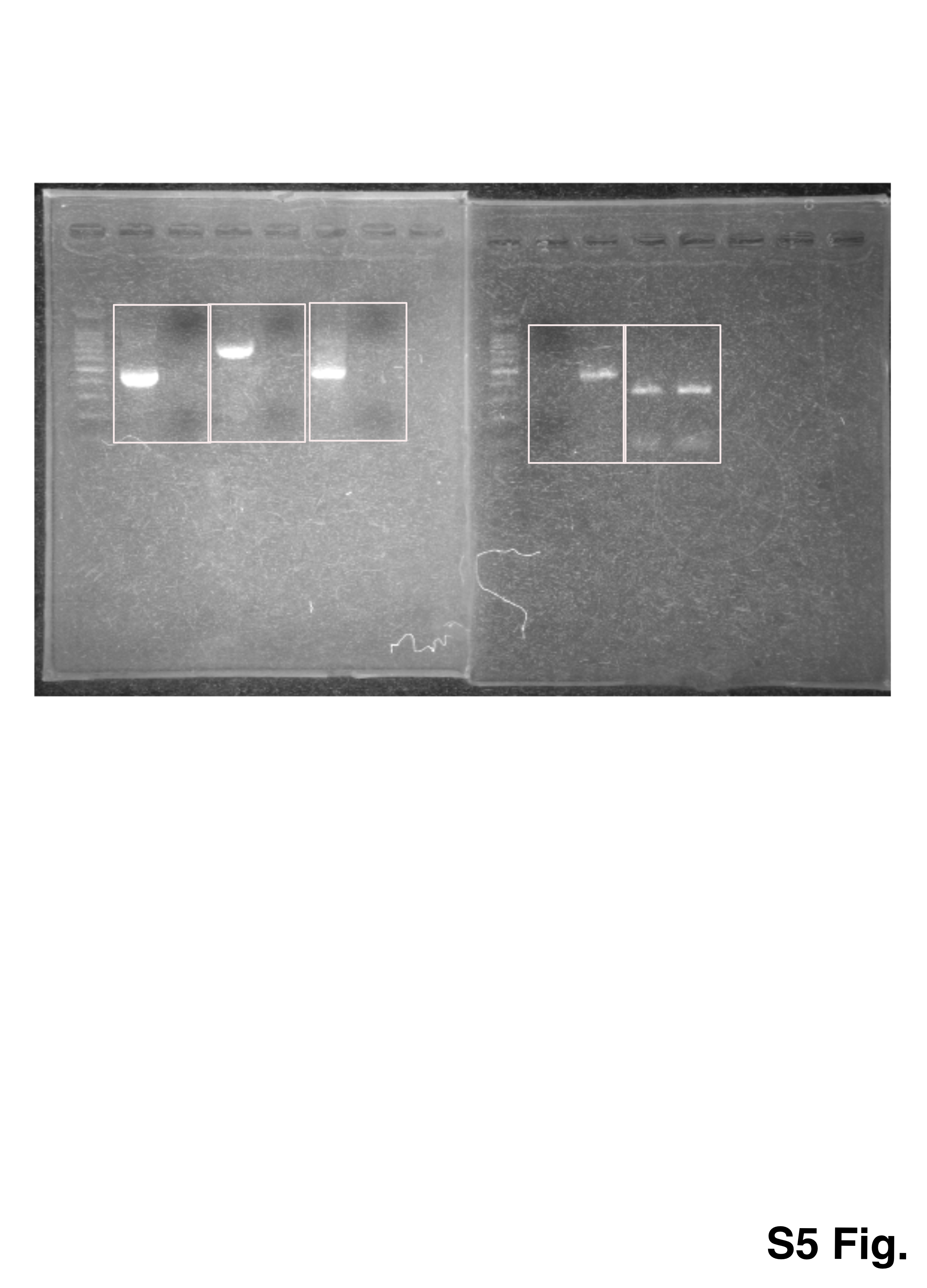

Supplement: S5 Fig — The corresponding area of the gel images were indicated by white rectangles. (TIFF) [file pone.0221364.s005.tiff]

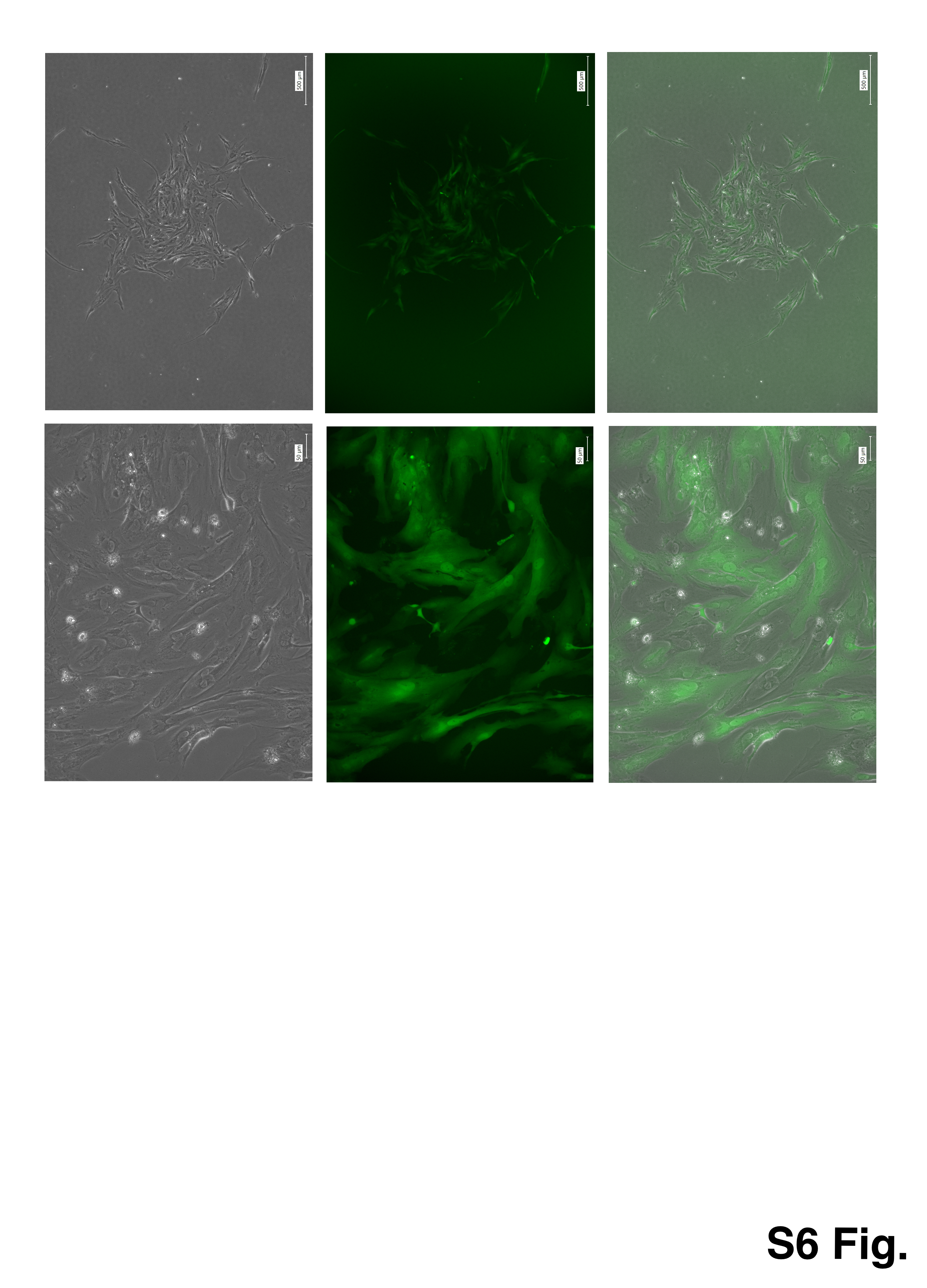

Supplement: S6 Fig — EGFP Fluorescence can be detected which come from the expression cassette. (TIFF) [file pone.0221364.s006.tiff]
